# Global variability in hydraulic traits and water use strategies of mountain shrubs and dwarf shrubs

**DOI:** 10.1111/plb.70088

**Published:** 2025-09-03

**Authors:** C. Musso, A. Ganthaler, S. Mayr

**Affiliations:** ^1^ Department of Botany University of Innsbruck Innsbruck Austria

**Keywords:** Embolism, frost drought, growth forms, hydraulic conductivity, hydraulic traits, water deficit, woody species

## Abstract

Shrubs are perennial, multi‐stemmed woody plants whose adaptation to stress factors allows them to colonise extreme habitats, including high elevations. Accordingly, shrubs are one of the most important growth forms in mountain regions, but their hydraulic properties are poorly understood. We conducted a literature search on the water use strategies of mountain shrubs, focusing on their main hydraulic traits related to water uptake, transport and release, as well as hydraulic limitations in summer and winter. In addition, the leaf cuticular conductance was measured in selected Alpine species. A total of 104 publications were found, mainly from North America, Europe and Asia, and a few from Africa and South America, with snow and steppe habitats strongly underrepresented. The dataset revealed a wide range of specific hydraulic conductivity (*k*_s_; 0.8–25.8 × 10^−4^ m^2^s^−1^ MPa^−1^), with highest values in tundra shrubs, and of the water potential at 50% conductivity loss (Ψ_50_; −11.8 to −0.29 MPa), with lowest values in steppe and temperate dry summer species. Deep‐rooted shrubs from arid environments had access to more reliable water sources, while others relied on shallow but nutrient‐rich soil water. No clear trend was observed along elevation or precipitation gradients, suggesting a wide range of hydraulic strategies to achieve a balanced water status. Shrub species from arid regions have to withstand low water potentials during the dry season, whereas temperate shrubs experience frost drought and freeze–thaw‐induced embolism in winter. The literature review revealed major gaps in the geographic distribution of available studies, and in our knowledge of root characteristics, recovery from embolism, and water storage capacity.

## Graphical abstract

Shrubs show a wide range of hydraulic strategies to achieve a balanced and favourable water status in mountain areas.

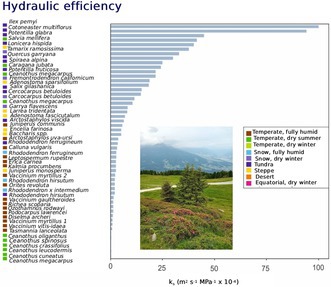

## INTRODUCTION

Shrubs are woody perennial species that are an important component of many ecosystems and can colonise extreme habitats, including high elevations and latitudes (Körner 2003; Myers‐Smith *et al*. 2015; Anadon‐Rosell *et al*. 2017). The growth form is characterised by a lower growth height compared to trees and a basitonic, sympodial growth with multiple independent stems, usually branching at the ground level (Beikircher & Mayr 2008). This growth habit allows shrubs to spatially expand faster than (young) trees, as they have a larger area for sprouting and can quickly produce branches and a canopy. This ensures their survival and growth despite the eventual loss of stems (Götmark *et al*. 2016). Shrubs with an extremely short growth height are recognised as dwarf shrubs (also known as “subshrubs”). The common definition of shrubs thus includes a wide variety of growth heights, from for example, 1 to 2‐m tall individuals in areas where the growing season is longer and winds are less strong, allowing them to grow taller into prostrate and upright dwarf and cushion shrubs in areas where strong winds and ice abrasion are common (Rundel 1991). The low growth height of dwarf shrubs helps them to avoid strong winds and reduce water loss through transpiration, which is beneficial in windy environments, such as at high elevations (Kemppinen *et al*. 2021). At the same time, in cold alpine environments, shrubs profit from their compact and low growth which creates a more favourable microclimate near the surface and ensures protection by snow cover in winter (Larcher 2012). This low growth also helps minimise mechanical stress caused by weight of the snowpack (Hacke *et al*. 2001). Shrubs and dwarf shrubs can colonise several habitats where trees cannot survive (Götmark *et al*. 2016), as their architecture, morphology, phenology, and physiology are very well adapted to different environments and to multiple stresses. These adaptations have made them ecologically dominant in warm and cold desert scrublands, Mediterranean shrublands, tropical alpine fields, heathlands, bogs, temperate forests, and tropical regions characterised by nutrient limitation, as well as in degraded shrublands, shrub savannas, oceanic islands, and the Arctic and Alpine tundra (Rundel 1991; Götmark *et al*. 2016). Shrubs are also an important component of the understorey vegetation (Carlquist & Hoekman 1985). This review focuses on shrubs from mountain regions, as these areas cover a significant proportion of the Earth's surface, provide essential ecosystem services, are particularly sensitive to climate change, but are also under‐represented in plant hydraulic studies (Grumbine & Xu 2021; Körner *et al*. 2021).

Climate conditions in mountain areas are shaped by an almost linear decrease in atmospheric pressure with increasing altitude, resulting in lower air temperatures, lower absolute air moisture content and lower vapour pressure. In contrast, UV rates and precipitation generally increase with elevation in temperate mountain areas, while in many subtropical and tropical mountains, precipitation decreases above a mid‐altitude maximum (Körner 2003). However, mountain plants experience their own microclimate or “bioclimate”, depending on their growth form and height, and on physical characteristics such as slope and exposure, which affect solar radiation, water availability and wind load (Larcher 2011). In general, the closer the plant is to the ground, the greater the decoupling of its microclimate conditions from atmospheric conditions. Low‐growing vegetation like dwarf shrub heaths warms up more than taller trees during the day under clear skies due to soil heat absorption but can lose more heat through radiation at night (Körner 2003; Larcher 2011).

Global climate change is expected to cause significant shifts in temperature and precipitation in mountain regions, as high latitude and high elevation regions warm faster than the global average (Hoegh‐Guldberg *et al*. 2018). This will affect alpine plant life, including shrubs, which are sensitive to changing climate conditions and can serve as bio‐climatic proxies (Myers‐Smith *et al*. 2015). An increase in shrub growth, expansion, and dominance has been observed globally, which is related to rising temperatures and changes in agricultural practices (Myers‐Smith *et al*. 2011). Shrub expansion and encroachment on former meadows can drastically alter the ecosystem carbon balance and potentially cause climate change feedbacks, with reductions in surface albedo and hydrological impacts (Maestre *et al*. 2016; Anadon‐Rosell *et al*. 2017; van den Bergh *et al*. 2018). In addition, shrub expansion may reduce the richness and diversity of alpine vegetation, highlighting the importance of studying its future dynamics (Rumpf *et al*. 2018; Grigoriev *et al*. 2022). Low atmospheric pressure in mountain regions increases the diffusion coefficient for water vapour, leading to increased transpiration (Smith & Geller 1979). This effect may be exacerbated by climate change because of changes in precipitation and temperature patterns. Shallow mountain soils make it difficult for plants to develop deep roots, making them more vulnerable to the effects of climate change and local precipitation (Bodé *et al*. 2020). Furthermore, earlier snowmelt, or even the lack of snow cover, can expose shrubs to freeze–thaw cycles. As climate change accelerates in mountain regions (Jones & Moberg 2003), understanding the hydraulic strategies of shrubs is important to predict shifts in ecosystem dynamics.

The water relations of woody plants growing in mountain areas vary considerably, depending on the environment, reflecting the high variability of habitat conditions at different spatial and temporal scales. Occasional water deficits may occur, depending on the amount of rainfall during the growing season, but also on the plant cover, soil structure and the associated water‐holding capacity (Körner 2003; Ganthaler & Mayr 2021). Mountain soils vary on a small scale and are “discontinuous over time” because changes in soil‐forming conditions can be sudden in relation to natural or human‐driven phenomena. Slopes enhance both vertical and lateral fluxes of water, dry matter and solutes, inducing intense dynamic processes leading to shallow and thick soils in erosion and accumulation areas, respectively (Egli & Poulenard 2016). Mountain soils are often loaded with raw humus in the upper soil layers, which increases the water‐holding capacity, while coarse rock fragments in the deepest layers reduce the water‐holding capacity. As a result, the thinner the humus layer and the coarser the debris in the soil, the less water will be available for plants, and the more vulnerable they become to extreme physical conditions (Körner 2003).

In winter, frost‐induced drought and freeze–thaw events threaten woody mountain plants, especially at higher elevations. Low winter temperatures freeze the soil and stem water for long periods, stopping the water supply, although transpiration continues to some extent (Mayr *et al*. 2020). These conditions cause a lowering of the water potential (Ψ), which can lead to the formation of winter embolism and impairment of the water transport system (Sparks & Black 2000; Mayr, Schwienbacher, *et al*. 2003). Frequent freeze–thaw cycles can intensify this phenomenon through the formation of air bubbles during freezing and their expansion upon thawing (Tyree *et al*. 1994; Pittermann & Sperry 2003). In this respect, snow cover can protect low‐growing plants by buffering low temperatures, preventing frost, shielding from intense solar radiation, limiting water losses through transpiration, and improving plant and soil water status during the snow melt in spring (Gerdol *et al*. 2013). However, one disadvantage of a prolonged snow cover is shortening of the growing period (Wipf *et al*., 2009). In contrast, in tropical mountain regions, the seasonal temperature variations are only moderate compared to the daily temperature fluctuations, but heavy rainfall often alternates with dry periods during the seasons (Sarmiento 1986).

Our knowledge of the ecophysiology of shrub species in general, and of mountain species in particular, is limited, possibly because of the overall lower economic value of this growth form compared to trees (Ganthaler & Mayr 2021). Despite their ecological role in many ecosystems worldwide, we have little information on shrub hydraulic characteristics and species‐specific drought resistance. In view of current global warming, changes in precipitation patterns and snow cover dynamics, as well as the abandonment of traditional land use leading to encroachment of formerly grazed mountain pastures (Jarque‐Bascuñana *et al*. 2022), improved insights into the water relations of mountain shrubs and dwarf shrubs will be a prerequisite for a better and more comprehensive understanding of mountain ecosystems under current and future conditions (Mayr *et al*. 2012, 2019).

In the following, we summarise current knowledge on the water relations of shrubs and dwarf shrubs in mountain areas, focusing on main hydraulic traits related to water uptake (e.g. rooting patterns), water transport (e.g. specific hydraulic conductivity, vulnerability to embolism, and xylem anatomical features), transpiration (e.g. stomatal regulation), as well as summer and winter limitations. Datasets based on the comprehensive literature review were complemented with new data on leaf cuticular transpiration of selected Alpine species. The main aims were to (1) summarise current knowledge on the hydraulic strategies of mountain shrubs, (2) describe variations in their hydraulic traits with respect to climate and habitat, (3) highlight possible differences from co‐occurring trees, and (4) identify current knowledge gaps.

## MATERIAL AND METHODS

### Literature search and analysis

The literature search was conducted in Web of Science™ (WOS; accessed 22 November 2024) with the option to search all databases (i.e. “Search in: All Databases”) to identify publications related to water relations of shrubs and dwarf shrubs from mountain areas. The literature search was inspired by Gurgiser *et al*. (2022), who aimed to identify most publications available on the WOS from 1900 to 2019 that were related to mountain systems. However, we did not limit the publication year field (i.e. PY) to any specific period. The string included general mountain terms, such as “alpine”, “mountain” or “montane”, as well as names of mountain ranges, and excluded some misleading terms. This was implemented in the WOS as:

TIORABORAK=mountain termsORrange namesANDgrowth formsANDhydraulic terms,

where, TI, AB and AK are the search title, abstract, and author keywords, respectively. The *mountain terms* in this string were “mountain” OR “alpine” OR “montane” OR “mountainous”; the *range names* correspond to the list of mountain ranges and sub‐ranges used in the literature review of Gurgiser *et al*. (2022); the *growth forms* were “shrub*” OR “chamaephyte” OR “erica”; while the *hydraulic terms* were “hydraulic*” OR “water relation*” OR “embolism” OR “drought” OR “transpiration” OR “water uptake”.

All the publications were manually reviewed. The inclusion criteria were, in order of importance, the target species (which had to belong to the shrub growth form, so each species was manually checked), the geographic origin of the research (i.e. the study area should be a mountain area), and the eco‐physiological topic (i.e. the publication provided information on plant water relations). Only publications that met these aims of the review were included for further analysis. We selected publications from mountain areas based primarily on the presence of mountain‐related terms or range names in their Abstract (as listed above). It is thus possible, that mountain‐related publications which used only geographic or elevation information in the title, abstract or keywords might have been overlooked, but according to previous search optimizations (Gurgiser *et al*. 2022), we expect the number of these publications to be small. Studies that included both shrubs and trees were only included if the hydraulic characteristics of these growth forms were clearly distinguished. These studies were particularly useful for comparing the hydraulic characteristics of shrubs and trees, which is one of the aims of this study. On the other hand, studies that focussed on the vegetation in general, without distinguishing between shrubs and trees, were excluded.

The Köppen‐Geiger digital world map for climate classification was used to categorise the studies (Kottek *et al*. 2006; an updated version was firstly assessed at: http://koeppen‐geiger.vu‐wien.ac.at/ on 5 December 2023 with a resolution of 5 arc minutes). We chose the Köppen‐Geiger classification system because it is one of the most widely used climate classifications based on temperature and precipitation patterns, it is commonly used to study ecological patterns (Theobald *et al*. 2024), and its digital version enables a rapid assignment for each study site. Based on the geographic coordinates, each study site was categorised on the digital world map according the annual and monthly temperature and precipitation means. In addition, the Climatic Research Unit (CRU) dataset from the University of East Anglia (Mitchell & Jones 2005; available at: http://www.cru.uea.ac.uk/) was used to obtain the mean annual precipitation (MAP, mm; 1961–90 normals) for study sites where this information was not available in the publication. For studies that reported more than one study area or that were conducted along an elevational gradient, each study site (and therefore the study species) was independently assigned to a climate zone. All studies were assigned to the five climate classes: equatorial (high temperature year‐round, e.g. Mount Kilimanjaro in equatorial Africa), arid (low precipitation, such as in mountain regions from desert areas, e.g. Arizona, the Qinghai‐Tibetan Plateau), warm‐temperate (moderate temperature and precipitation, e.g. the Western Cordillera, North America), snow (e.g. significant temperature fluctuation between summer and winter, such as the Sierra Nevada mountain range), or polar (high elevations, precipitation mostly in the form of snow, e.g. Andes) according to the five vegetation groups of Köppen (Kottek *et al*. 2006).

### Cuticular transpiration of Alpine dwarf shrubs

Measurements of cuticular transpiration (g_min_, mmol m^−2^ s^−1^) were performed according to the protocol of Sack & Scoffoni (2010) on 7–8 plants per dwarf shrub species, as independent biological replicates, harvested on Mt. Patscherkofel (1883 m a.s.l.; Tyrol, Austria, 47°22′N, 11°47′ E), namely *Arctostaphylos uva‐ursi* (L.) Spreng., *Rhododendron ferrugineum* (L.), *Kalmia procumbens* (L.) Gift & Kron, and *Vaccinium vitis‐idaea* (L.). First, twigs were water‐saturated (Ψ ≥ −0.1 MPa), then leaves were dehydrated using a fan (i.e., average wind speed 1.9 m s^−1^). During dehydration, temperature and relative humidity were monitored using common temperature–humidity sensors. At intervals, leaves were sealed in plastic bags to prevent water loss before their weight was measured. The g_min_ was calculated from differences in leaf weight over time, air temperature, relative humidity, and mean leaf area.

### Statistics

Average values are given as mean ± SE. Significant differences between hydraulic parameters (*k*_s_ and Ψ_50_; Table 2) from different climate zones, and cuticular conductance (g_min_; Table 3) between dwarf shrub species were tested using a One‐way ANOVA, followed by a Tukey post‐hoc test in R (functions “aov” and “TukeyHSD” in package “stats”; R Core Team 2021). Relationships between elevation, mean annual precipitation and hydraulic parameters (*k*_s_ and Ψ_50_; Fig. 5) were tested using a linear model (“lm()”) function in the R package “stats”, where the predictor (independent variable) was the logarithm of elevation or mean annual precipitation, and the dependent variable was either *k*_s_ or Ψ_50_.

## RESULTS

**Fig. 1:**
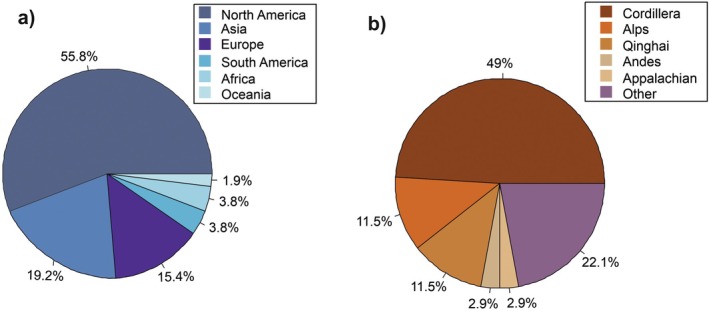
Proportion (%) of studies on mountain shrubs according to (a) geographic areas and (b) mountain regions of the study sites. Mountain regions included the North American Cordillera (Cordillera), the Alps (Alps), the Qinghai‐Tibetan Plateau (Qinghai), the Andes (Andes), the Appalachian Mountains (Appalachian), and several other areas that were represented by only a few studies (Other).

**Fig. 2:**
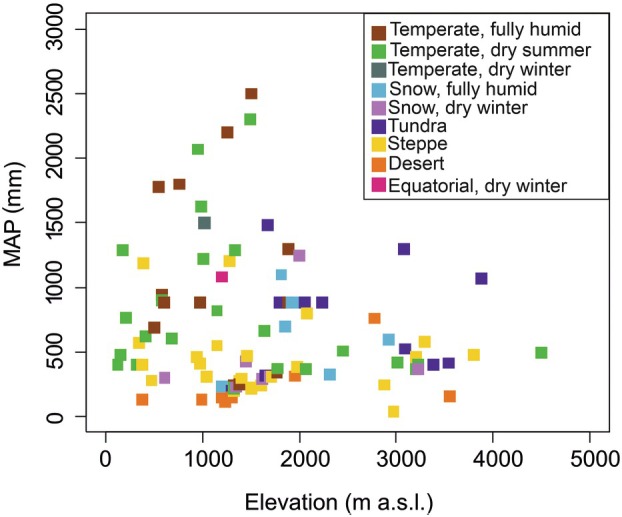
Mean annual precipitation (MAP; mm) as a function of elevation (m a.s.l.). Each data point represents a study area, coloured according to the Köppen‐Geiger climate classification. The nine classes refer to five main climates with subcategories: warm temperate (Temperate, fully humid; Temperate, dry summer; Temperate, dry winter), snow (Snow, fully humid; Snow, dry winter), polar (Tundra), arid (Steppe; Desert) and equatorial (Equatorial, dry winter).

The literature search resulted in 1564 publications covering 140 species (for full species list see Table S1). A large proportion of these publications were excluded because they did not relate to mountain systems, their target species were not shrubs, or they did not report any water relations parameters. Finally, the review was based on 104 publications, most of them from North America (number of studies, *n* = 58), followed by Asia (*n* = 20), Europe (*n* = 16), South America (*n* = 4), Oceania (*n* = 2) and Africa (*n* = 4; Fig. 1a). For the mountain ranges covered in this review, most of the studies were from the North American Cordillera (*n* = 51), the Qinghai‐Tibetan Plateau (*n* = 14) and the Alps (*n* = 12; Table 1). There were also a few publications from the Andes (*n* = 3) and the Appalachian Mountains (*n* = 3; Fig. 1b), and several studies were from mountains not belonging to any of the defined mountain ranges (*n* = 21). The mean annual precipitation on analysed study sites ranged from 42 to 3650 mm, while elevational range covered areas from 126 to 4500 m a.s.l. (Fig. 2).

**Table 1:** Summary of selected studies by mountain range (mountain), indicating the number of studies (number of studies), mean annual precipitation (MAP, mm), and elevation range (elevation m a.s.l.).

mountains	number of studies	MAP (mm)	elevation (m a.s.l.)
Cordillera	51	115–2500	126–3801
Alps	12	692–1951	500–2236
Qinghai‐Tibetan Plateau	14	42–580	1712–4500
Andes	3	160–1300	3079–3877
Appalachian	3	1083–1800	760–1198
Other	21	218–3650	345–3800

The studies analysed were published in 50 journals, with *Global Change Biology* having the highest Journal Citation Indicator (JCI) 2022. In total, 20 journals (40%) had a JCI >1. Web of Science (WOS) classified (i.e. doctype) all studies as articles, except for one review (Jacobsen & Pratt 2014). The years of publication ranged from 1974 to 2024, and the field of all publications was “Science & Technology”. In total, we found 10 WOS subjects, with the most frequent one being “Ecology” (35%), followed by “Plant Sciences” (28%) and “Environmental Sciences” (14%); 13 publications (26%) had more than one subject category. The number of publications shows a steady increase over the years, with significant growth in the 1990s and 2000s, and a notable peak in 2022.

### Water uptake

The characteristics of the rooting system and the root growth dynamics of mountain shrubs are still largely unresolved. Alpine plants typically have deep roots, often reaching 1 m in depth, to access water when the surface soil dries out, with dwarf shrubs having a higher proportion of belowground biomass than forbs (Körner 2003). Understanding how shrubs use water in mountains worldwide can help to estimate the amount of transpiration and evapotranspiration in these areas (e.g. Bailey *et al*. 2022). It also helps to study how competition for water resources can outcompete species using the same water resource (e.g. Bonham & Mack 1990; Darrouzet‐Nardi *et al*. 2006). Additionally, it reveals adaptive strategies, such as the development of lateral roots in drier mountain areas, which help shrubs to thrive (Gibbens & Lenz 2001). Insights into the water use of mountain shrubs provide a comprehensive overview of the morphology, growth and depth of root systems, especially in mountain soils where cracks and cervices may occur (e.g. Rong *et al*. 2011). Furthermore, using the stable isotopes of water to understand from which soil layers shrubs take up water determines how changes in the isotopic composition of precipitation alter the water uptake strategy of shrubs (e.g. in tropical mountains; Bodé *et al*. 2020).

Environmental factors, such as soil moisture, properties, depth and temperature, strongly influence water uptake in mountain shrubs. Zhang *et al*. (2022) demonstrated this in a study of eight subalpine shrub species, showing that water sources used during periods of water limitation varied with aspect (semi‐sunny and semi‐shaded). Many of the shrub species studied were able to adapt their water uptake to different water sources (Dodd *et al*. 1998; Darrouzet‐Nardi *et al*. 2006), and the preferred use of shallow or deep water has been interpreted as an advantageous strategy, especially in the case of competition for water resources (Ehleringer & Dawson 1992; Wu *et al*. 2016; Zhang *et al*. 2020, 2022; Shi *et al*. 2021). The preferred use of shallow water by some mountain shrubs could also be related to the higher concentration of nutrients in this soil layer or to its generally high water‐holding capacity (Ehleringer & Dawson 1992; Dudley *et al*. 2018; Wu *et al*. 2019a). Access to deep water reservoirs through deep roots (characteristic of woody species from dry sites; Fernandez & Caldwell 1975; Franco *et al*. 1994; Dodd *et al*. 1998; Gibbens & Lenz 2001; Schwinning *et al*. 2005; Gonzalez‐Rodriguez *et al*. 2017; Szutu & Papuga 2019; Zhu *et al*. 2021) may help other shrub species to access more reliable deep‐water sources during drought (e.g. access groundwater through deep roots) (Dawson & Pate 1996; Sternberg *et al*. 1996) and, in general, in drier environments.

Stable isotopes of water (δ^18^O, δ^2^H) can be used to determine the source of water uptake by plants and to estimate rooting depth (Ehleringer & Dawson 1992; Dawson *et al*. 2002). The isotopic signature of xylem water can be directly compared with that of soil water at different depths or, more generally, to potential water sources, as there is no significant isotopic fractionation during soil water uptake and transport through the plant xylem (Lambers & Oliveira 2019). Our literature search identified 24 publications on 48 mountain shrub species that used stable isotopes of water to investigate patterns of rooting depth in mountain areas. Many of the species were able to use different water sources, depending on the availability of rainwater (e.g. Li *et al*. 2007; Wu *et al*. 2016; Wen *et al*. 2022), season (e.g. Donovan & Ehleringer 1994; Wu *et al*. 2019b; Bodé *et al*. 2020; Zhang *et al*. 2020; Bailey *et al*. 2022), growing season stage (Tian *et al*. 2023), and depth of the water table (e.g. Chimner & Cooper 2004). Mountain shrubs were found to have mostly dimorphic rooting systems (e.g. Duan *et al*. 2008; Zhu *et al*. 2016, 2021; Zhang *et al*. 2022; Tian *et al*. 2023), although some species preferentially used water from either shallow or from deeper soil layers (e.g. Thorburn & Ehleringer 1995; West *et al*. 2012; Dudley *et al*. 2018). In particular, the use of deep‐water resources has been shown to be a common and advantageous strategy to be competitive under water limitation, especially for co‐occurring species. Some studies (Kitajima *et al*. 2013; Prieto & Ryel 2014; Zhu *et al*. 2021) also documented the redistribution of water by hydraulic lift during the dry season.

Several studies suggested competition between co‐occurring mountain shrubs and trees for the same water resources. However, a recent study (Goodwin & Hurteau 2024) found that the presence of shrubs had no effect on the water source dynamics of co‐occurring conifer trees. In addition, Kropp *et al*. (2016) showed that the canopy shading effect of trees may even be beneficial for some shrubs, such as *Larrea tridentata*, which grew roots mainly in the shallow soil layers. Sternberg *et al*. (1996) and Kitajima *et al*. (2013) found that trees and shrubs in the Californian mountains rely on deep‐water sources from the weathered bedrock, which may be advantageous given the low water‐holding capacity of shallow montane soils and dry seasons. Similarly, in a mountain forest environment in north‐eastern Mongolia, *Larix sibirica* and the woody shrub *Potentilla fruticosa* competed for water from similar depths (Li *et al*. 2007). Wen *et al*. (2022) compared the water sources of one tree and two shrubs in a *Pinus taiwanensis* Hayata community in the subtropical mountains and found that all species took water from deep soil layers (40–80 cm depth). However, during the rainy season and when the surface soil was waterlogged, these shrubs could also use water from upper soil layers (0–20 cm). Similarly, the introduced tree species *Robinia pseudoacacia* L. and two native shrubs in the Taihang Mountains (China) also compete for their water resources (Zhu *et al*. 2021). Specifically, this tree and one shrub species (*Ziziphus jujuba* Mill var. *spinosa*) used deep water (40–50 cm depth). The other shrub (*Vitex negundo* L. var. *heterophylla*) relied mainly on water from shallow soil horizons, although it switched to deeper layers during the dry season through its dimorphic rooting system. Another publication by Zhang *et al*. (2020) analysed the stable isotopic composition of oxygen in a shrub and a dwarf shrub species. The shrub species, *Caragana korshinskii*, from the mountains of Lanzhou city, was more flexible in its water uptake, shifting quickly between water sources, e.g. after a precipitation event. The dwarf shrub species (*Reaumuria soongorica*) could also vary uptake between shallow and deeper soil layers, but not as rapidly as *C. korshinskii*.

It is also important to consider developmental differences, as demonstrated by Donovan & Ehleringer (1994). These authors found that young, but not adult, *Chrysothamnus nauseosus* shrubs used water from different soil layers in response to seasonal drought. In contrast, Wu *et al*. (2019a) described that adult *Hippophae rhamnoides* shrubs could change the depth of water uptake under water limitation, while seedlings and juveniles mainly accessed water from the uppermost soil layers (Donovan & Ehleringer 1992).

The shallow and rocky nature of montane and alpine soils, with their limited water‐holding capacity, may be a challenge for shrubs under climate change (Bodé *et al*. 2020). Changes in precipitation patterns, such as reduced rainfall and changes in the timing and intensity of precipitation, or simply increases in temperature leading to higher evapotranspiration, may result in less frequent replenishment of soil moisture and potentially increased risk of drought stress for shrubs. In contrast, encroachment of shrubs and other woody plants was observed in many alpine and subalpine ecosystems worldwide (Komac *et al*. 2013). Furthermore, earlier snowmelt, which is becoming common under climate change, alters soil water availability and may lead to water deficits later in the growing season, posing additional challenges for shrub water uptake in mountain regions. Research on rooting patterns and growth dynamics (Bailey *et al*. 2022) thus is crucial to understand the impact of shrub encroachment on ecosystems and to develop management practices.

### Water transport

The characteristically low growth height of shrubs, as well as their contrasting growth pattern, implies different requirements for their hydraulic architecture compared to trees (Tyree & Ewers 1991; Ganthaler & Mayr 2015). Unlike trees, which typically have a single vertical stem with recognisable horizontal branches, shrubs have multiple shoots with side branches. This results, for example, in shorter transport distances in shrubs than in trees, different hydraulic resistances, and opposite morphological and anatomical characteristics (Beikircher & Mayr 2008).

The xylem supports long‐distance water transport in plants, accounting for 99% of the total length of the water pathway from roots to leaves and 50% of total plant hydraulic resistance (Nardini *et al*. 2011). The extra‐vascular compartment contributes to another 50% of the resistance with only 1% of the length. Shrubs, like all plants, require a water transport system that is both sufficiently efficient and sufficiently safe, and the observed differences between plant species likely reflect their ecology and evolution (Gleason *et al*. 2016). In the following section, we review existing knowledge of the physiological and anatomical traits related to water transport in mountain shrubs.

#### Xylem hydraulic efficiency

Specific hydraulic conductivity (*k*_s_) is a measure of the efficiency of water transport in the xylem (Tyree & Ewers 1991). Conductivity is expected to increase with the size and number of conduits per unit stem cross‐sectional area and with decreasing pit resistance (Sperry *et al*. 2008). Leaves are well supplied with water when the *k*_s_ is high, transport distances are short, and there is a high proportion of conductive cross‐sectional area per leaf area.

**Fig. 3:**
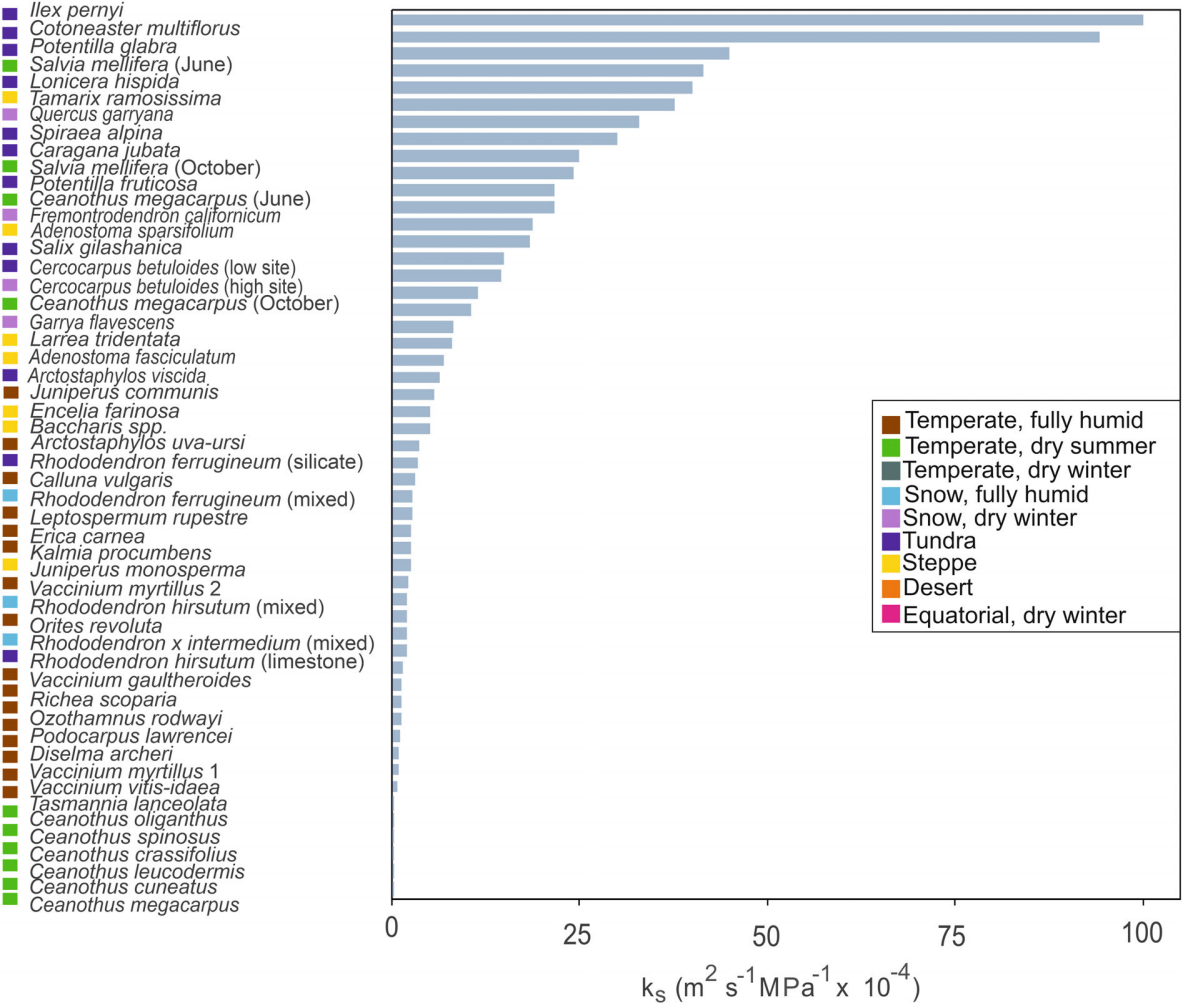
Specific hydraulic conductivity (*k*_s_) for all mountain shrub species found in the literature search. Values were partly extracted from published figures. For *Vaccinium myrtillus*, two studies were available (indicated by numbers, Ganthaler & Mayr 2015, Ganthaler *et al*. 2022), and for some species several values measured during the season, on different soil types, or at different elevation (e.g. high, low) were available (indicated in brackets). Coloured boxes indicate the Köppen‐Geiger climate classification (see Fig. 2, no *k*_s_ values were available for the Temperate, dry winter, Equatorial, dry winter and Desert climates).

Analysed publications covering 45 species revealed that most shrubs from mountain areas have a *k*_s_ between 0.8 and 25.8 × 10^−4^ m^2^s^−1^ MPa^−^1, with an overall average of 13.3 ± 3.5 × 10^−4^ m^2^s^−1^ MPa^−1^. However, extremely low (Davis, Sperry, *et al*. 1999) as well as high *k*_s_ values up to 100 × 10^−4^m^2^s^−1^ MPa^−1^ were also reported for some semi‐ring porous shrub species (Feng *et al*. 2022). Shrubs from tundra climates had the highest *k*_s_ values, while those from temperate regions tended to have the lowest (Figs. 3 and 5a, Table 2). Notably, some of the latter shrubs grow in the understorey and may exhibit low *k*_s_ due to the reduced transpiration rates and lower risk of embolism formation under low light conditions (Carlquist & Hoekman 1985). No clear trend between *k*_s_ and elevation was found (Fig. 5c; *R* ^2^ = 0.14). The observed mean hydraulic efficiency is comparable to the mean *k*_s_ reported in the Xylem Functional Traits Database (https://xylemfunctionaltraits.org; Gleason *et al*. 2016) for shrub species, averaging 16.6 ± 0.1 × 10^−4^m^2^s^−1^ MPa^−1^. In mountain shrubs, *k*_s_ values differ between deciduous angiosperm shrubs (22 ± 7.1 × 10^−4^m^2^s^−1^ MPa^−1^), evergreen angiosperm shrubs (9.9 ± 2.4 × 10^−4^ m^2^s^−1^ MPa^−1^), and evergreen conifer shrubs (2.6 ± 0.9 × 10^−4^m^2^s^−1^ MPa^−1^). Similar differences between deciduous and evergreen shrubs were also reported by Maherali *et al*. (2004).

**Table 2:** Mean specific hydraulic conductivity (*k*_s_, m^2^s^−1^ MPa^−1^ × 10^−4^) and mean vulnerability to xylem embolism (water potential at 50% loss of conductivity; Ψ_50_, MPa) for mountain shrubs from different climate regions, classified according to Köppen‐Geiger (mean ± SE).

Köppen‐Geiger climate	conductivity		vulnerability	
	*k*_s_	N° species	Ψ_50_	N° species
Temperate, fully humid	2.1 ± 0.3 a	16	−2.5 ± 0.3 b	11
Temperate, dry summer	9.8 ± 4.4 b	10	−6.5 ± 0.8 ab	10
Snow, fully humid	2.2 ± 0.2 a	3	−2.9 ± 0.6 ab	3
Snow, dry winter	17.8 ± 4.8 ab	4	−2.5 ± 0.8 b	3
Tundra	33.1 ± 8.1 ab	12	−3.2 ± 0.4 a	5
Steppe	11.9 ± 4.4 ab	7	−5.2 ± 0.6 ab	15

Statistically significant differences between climate regions (*P* < 0.05) are indicated with different letters. Significant differences were tested using a One‐way ANOVA followed by a Tukey post‐hoc test in R (R Core Team 2021).

The average *k*_s_ of the analysed shrubs was significantly lower than that of trees, which average 28.4 ± 0.2 × 10^−4^m^2^s^−1^ MPa^−1^ according to the Xylem Functional Traits Database (Gleason *et al*. 2016). One study comparing shrub and tree growth forms of the same species, *Juniperus communis*, found that shoots of shrub‐like individuals were similar to tree branches in morphology and hydraulic efficiency, while tree stems had a significantly higher *k*_s_ (Beikircher & Mayr 2008). Similarly, interspecific studies comparing co‐occurring shrubs and trees found that the mean *k*_s_ of shrubs was generally lower than that of trees (Gartner 1991; Tyree & Ewers 1991; Pockman & Sperry 2000; Ganthaler & Mayr 2015; Ganthaler *et al*. 2022). These results suggest that mountain shrubs, regardless of geographic location, tend to have lower hydraulic efficiency than trees, possibly related to their smaller height.

The dataset also included six dwarf shrub species (Ganthaler & Mayr 2015; Ganthaler *et al*. 2022), a rarely studied group of plants. The *k*_s_ of these species averaged 2.1 ± 0.3 × 10^−4^m^2^s^−1^ MPa^−1^, which was lower than the mean *k*_s_ of the remaining shrub species in the dataset (15.3 ± 4.0 × 10^−4^ m^2^s^−1^ MPa^−1^). The relatively low *k*_s_ of these Alpine dwarf shrub species was explained by a relatively high proportion of non‐conducting xylem elements (19–49%; Ganthaler *et al*. 2022). Given the strong winds, snow loads, and generally harsh conditions in which these dwarf shrubs thrive, they can survive despite their low *k*_s_ because their small height helps create a favourable microclimate, with a low‐water vapour pressure deficit in the canopy. As a result, they might not need high transport capacities and can optimise their xylem for other requirements, such as mechanical loads.

Stem and leaf hydraulic efficiencies of shrubs appear to decrease with increasing elevation, as found in a study by Feng *et al*. (2022) that analysed nine mountain shrubs along an elevational gradient to the treeline. Similarly, the mean *k*_s_ of riparian shrub species was found to be overall higher than that of upland shrubs (15.8 ± 0.001 compared to 5.2 ± 0.0001 × 10^−4^m^2^s^−1^ MPa^−1^; Pockman & Sperry 2000). In contrast, Davis, Ewers, *et al*. (1999) and Davis, Sperry, *et al*. (1999) found no changes in *k*_s_ when comparing two subgenera of *Ceanothus* along an elevation gradient from 280 to 1100 m a.s.l. in California. However, the specific hydraulic conductivity of these species from arid Mediterranean environments is overall very low (compare Fig. 3), suggesting that in dry mountains, shrubs may have lower *k*_s_ values to favour hydraulic safety (i.e., more negative Ψ at 50% loss of conductivity, see “*Xylem hydraulic safety*”).

#### Xylem hydraulic safety

The safety of xylem transport is determined by the xylem water potential, which induces a significant loss of hydraulic conductivity because of embolism formation (Gleason *et al*. 2016). So‐called vulnerability curves show the relationship between conductivity loss and water potential. The water potential inducing 50% loss of conductivity (Ψ_50_, MPa) is used to characterise the species‐specific hydraulic safety and provide information on drought resistance of a species (Tyree & Ewers 1991; Pockman & Sperry 2000; Choat *et al*. 2012; Gleason *et al*. 2016), and together with the water potential at which plants lose 88% of maximum conductivity (Ψ_88_, MPa) describes the likelihood of drought‐induced hydraulic failure (Choat *et al*. 2012).

**Fig. 4:**
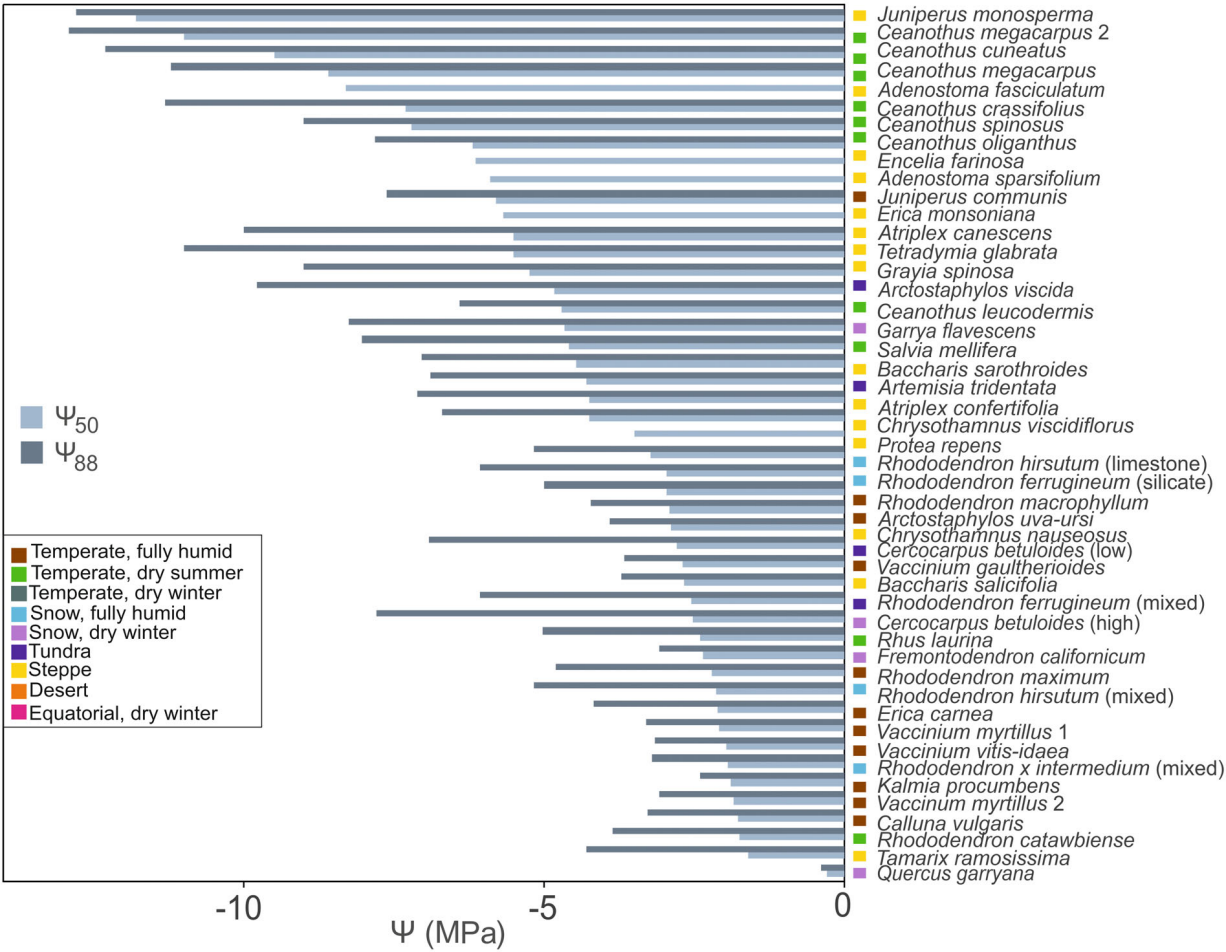
Xylem vulnerability thresholds representing water potential at 50% (Ψ_50_, MPa) and 88% (Ψ_88_, MPa) loss of conductivity for all mountain shrub species found in the literature search. For *Vaccinium myrtillus* and *Ceanothus megacarpus* two studies were available (indicated by numbers; Ganthaler & Mayr 2015, Ganthaler *et al*. 2022 for *V. myrtillus*; Kolb & Davis 1994 and Davis, Ewers, *et al*. 1999; Davis, Sperry, *et al*. 1999 for *C*. *megacarpus*) and for some species several values measured during the season on different soil types, or at different elevation (e.g. high, low) were available (indicated in brackets). For the species *Encelia farinosa*, *Erica monsoniana*, *Protea repens*, *Adenostoma fasciculatum* and *Adenostoma sparsifolium*, only Ψ_50_ was available. Coloured boxes indicate the Köppen‐Geiger climate classification (see Fig. 2; no Ψ_50_ and Ψ_88_ values were available for the Temperate, dry winter, Equatorial, dry winter or Desert climates).

**Fig. 5:**
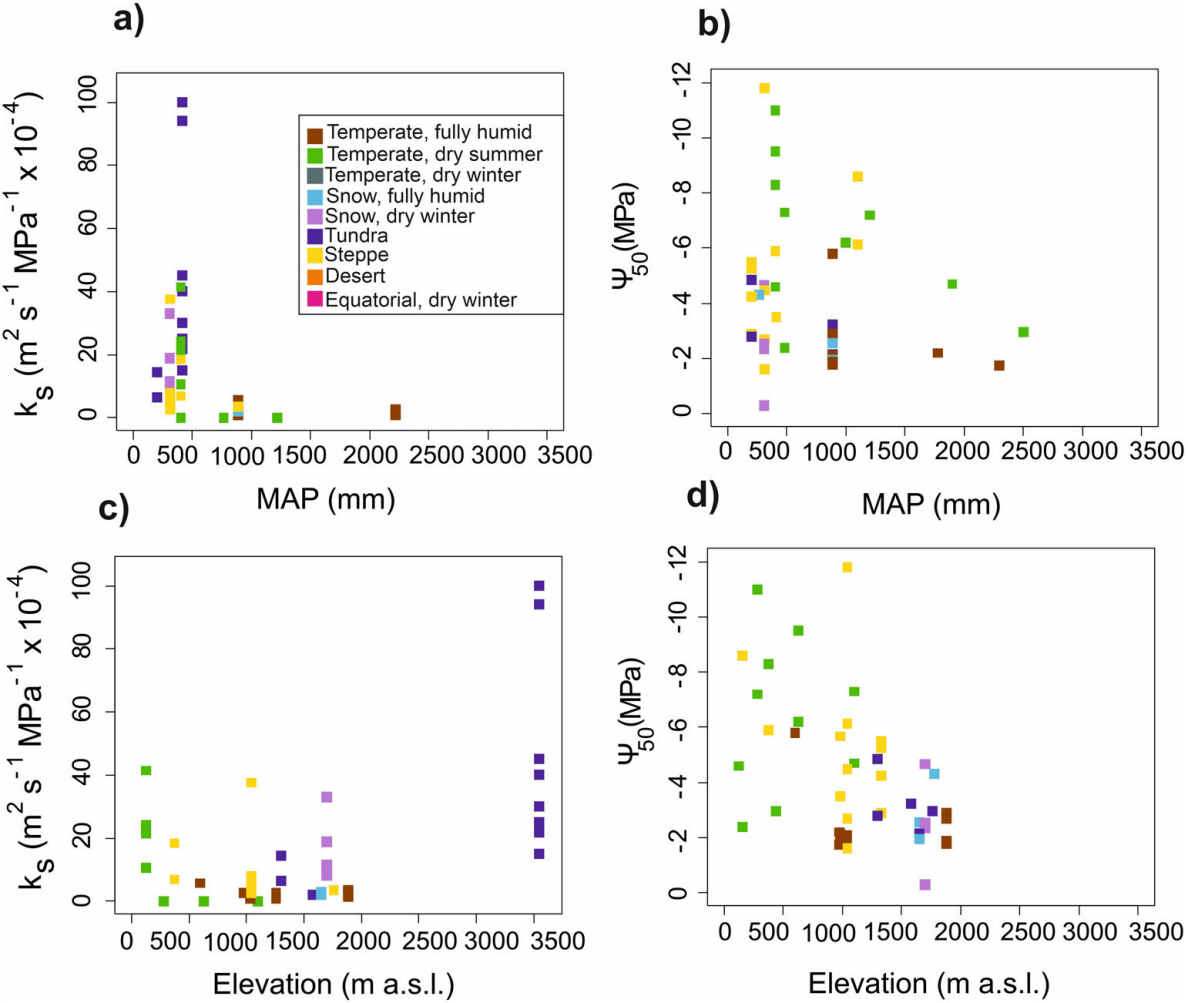
Specific hydraulic conductivity (*k*_s_) and embolism resistance (Ψ_50_) as a function of mean annual precipitation (MAP; a, b) and elevation (c, d) of the study site. Each data point represents one mountain shrub species, coloured according to the Köppen‐Geiger climate classification (see Fig. 2; no *k*_s_ and Ψ_50_ values were available for the Temperate, dry winter, Equatorial, dry winter or Desert climates).

The Ψ_50_ values obtained for 46 mountain shrub species varied from −11.8 to −0.29 MPa, with an average of −4.2 ± 0.4 MPa (Fig. 4). Most shrubs had a Ψ_50_ between −6.7 and −1.6 MPa, with exceptionally resistant species below this range either being conifers (*Juniperus*) or occurring in designated arid environments. Shrubs from steppe and temperate regions with dry summer climates had the most negative Ψ_50_ values, while species from temperate regions with fully humid climates tended to have less negative values (Fig. 4, Table 2). This indicates that shrubs in drier mountain regions require higher resistance to water stress to persist in these environments. No clear trend between Ψ_50_ and elevation has been observed (Fig. 5d; *R* ^2^ = 0.22). In contrast to our dataset, resistance to embolism can also vary with elevation, as in the studies of Davis, Ewers, *et al*. (1999), Davis, Sperry, *et al*. (1999) and Yao *et al*. (2024), who found that vulnerability to embolism increases with elevation. According to a meta‐study by Maherali *et al*. (2004), including deciduous, evergreen, and conifer species, shrubs were generally more resistant than trees. Accordingly, average Ψ_50_ values from the Xylem Functional Traits Database, with means of −3.9 ± 0.1 MPa for shrubs and −3.3 ± 0.07 MPa for trees (Gleason *et al*. 2016), also support the higher resistance of shrubs. Observations on *Juniperus communis*, growing as both tree and shrub, showed that the vulnerability of shrub shoots was comparable to that of tree stems and upper branches (Beikircher & Mayr 2008). Because these are shorter in height, they are less prone to embolism formation from drought or freezing, as the risk of cavitation increases with height of the stem (Ryan & Yoder 1997; Götmark *et al*. 2016).

Based on our dataset on mountain shrubs, the mean Ψ_50_ for evergreen angiosperm shrubs was −6.8 ± 0.7 MPa and −4.0 ± 0.4 MPa for the deciduous species. Only two conifer shrub species were included, with mean Ψ_50_ of ‐8.8 ± 2.1 MPa. These values are comparable to those reported by Maherali *et al*. (2004), who observed Ψ_50_ values of −5.1 ± 0.5, −4.5 ± 0.6 and −9.0 ± 1.8 MPa in North American evergreen angiosperms, deciduous angiosperms, and evergreen conifers, respectively. However, the hydraulic properties of mountain shrubs may vary with geological, topographical, or soil characteristics. For example, Alpine *Rhododendron* species exhibit different vulnerabilities when growing on silicate, limestone, and mixed soils (Mayr *et al*. 2010). Cordero & Nilsen (2002), analysing three *Rhododendron* species in North America, reported overall similar Ψ_50_ to rhododendrons from the Alps, where the most resistant species grow in arid areas (*R. hirsutum*, *R. macrophyllum*). Jacobsen & Pratt (2014) showed that chaparral shrub species from humid areas are more vulnerable than species from dry chaparral areas. Similarly, upland shrubs from a xeric site had lower vulnerability and larger safety margins (i.e. difference between minimum field water potential and Ψ_50_) than riparian shrubs (Pockman & Sperry 2000).

So far, the vulnerability of dwarf shrubs to drought‐induced embolism has only been analysed for species from subalpine dwarf shrub heaths in the European Alps. Results for species such as *Vaccinium myrtillus, Calluna vulgaris* and *Arctostaphylos uva‐ursi* by Ganthaler & Mayr (2015, 2021) showed an overall lower resistance than for conifer trees and shrubs from the same habitat (Mayr *et al*. 2006), but a Ψ_50_ close to co‐occurring *Rhododendron* shrubs. In addition, it seems likely that these dwarf shrubs avoid low water potentials through characteristic root and leaf functional traits, rather than withstanding them (Ganthaler & Mayr 2015, 2021).

Several studies have found a trade‐off between xylem hydraulic efficiency and safety in mountain shrubs (i.e. Kolb & Davis 1994; Kolb & Sperry 1999; Hacke *et al*. 2000; Pockman & Sperry 2000). Thus, protection against embolism formation seems to be associated with a lower *k*_s_. For example, *Salvia mellifera* had twice the *k*_s_ of the co‐occurring chaparral shrub *Ceanothus megacarpus* but was more vulnerable to xylem cavitation (50% loss of conductivity at −4.5 MPa compared to −11 MPa, respectively), possibly because the former is drought‐deciduous and the latter is evergreen (Kolb & Davis 1994). Furthermore, several studies on shrubs (Kolb & Sperry 1999; Mayr *et al*. 2010; Prieto & Ryel 2014) observed a correlation between Ψ_50_ and xylem conduit diameter, with larger (and longer) conduits being less resistant to drought‐induced embolism.

Two studies have reported the survival and recovery of shrubs from embolism. Mayr *et al*. (2019) highlighted the ability of the mountain shrub *Pinus mugo* to recover from embolism in late winter and spring. This recovery process was associated with changes in pit aspiration and carbohydrate content. The percentage of aspirated pits decreased with hydraulic recovery, while the change in carbohydrate content may have a role in xylem refilling. Additionally, Ganthaler & Mayr (2015) observed recovery from drought‐induced embolization in two mountain dwarf shrub species of the genus *Vaccinium*. These shrubs showed rapid embolism removal under favourable water conditions, possibly facilitated by their lower growth height and shorter hydraulic distances.

#### Xylem anatomical features

Xylem hydraulics are closely related to xylem anatomical traits. For instance, wider conduits lead to higher *k*_s_ but may lead to a higher risk of embolism formation during freeze–thaw cycles (Sperry & Sullivan 1992; Mayr *et al*. 2006; Pittermann & Sperry 2006). Pit architecture is also a major determinant of hydraulic efficiency and safety (Tyree & Zimmermann 2002). According to the rare pit hypothesis, the vulnerability to embolism is influenced by the number of pits in the conduit. Larger conduits with more pits are more likely to have at least one pit that is prone to air‐seeding (Wheeler *et al*. 2005; Hacke *et al*. 2006; Sperry *et al*. 2007; Venturas *et al*. 2017).

The mean conduit diameter of mountain shrub species in the dataset was 13.6 ± 1.2 μm, and the mean hydraulic diameter (diameter of conduits reflecting average hydraulic conductivity; Tyree & Zimmermann 2002) was ca. 17.5 ± 1.5 μm. These values, based on 16 species, are lower than the reported 25.2 ± 0.7 μm and 31.8 ± 1.2 μm in the Xylem Functional Traits Database (Gleason *et al*. 2016) for shrubs from all environments. *Juniperus communis* and *Rhododendrons* species (*R. ferrugineum*, *R. hirsutum*, *R. × intermedium*) from the Alps had the lowest mean conduit and hydraulic diameters, with 9.5 and 13.4 μm in the former, and 6.7 and 14.7 μm in the latter. Overall, the mean conduit and hydraulic diameters of mountain shrubs were lower than those of trees (31.7 ± 1.1 μm and 47.9 ± 2.8 μm, respectively; Xylem Functional Traits Database; Gleason *et al*. 2016).

The main function of the xylem is long‐distance water transport, but the xylem also provides mechanical support to the plant and serves as a storage site for water and carbohydrates. These functions are interrelated, resulting in trade‐offs (Pratt & Jacobsen 2017; Pratt *et al*. 2021). For shrubs and dwarf shrubs, mechanical support may be less important than for trees because of their smaller size. However, environmental stresses, such as wind, snow or avalanches, may require additional (mechanical) investment and potentially mask the relationship between hydraulic and anatomical traits. In addition, living cells in the xylem may be critical for the storage of non‐structural carbohydrates (NSCs; Gargiulo *et al*. 2024) especially in mountain regions with long winters.

### Transpiration

Transpiration controls water and nutrient uptake as well as evaporative leaf cooling, and its regulation can be critical for shrub survival in mountain conditions, which can include drought and heat stress. Moreover, studying the transpiration rates of mountain shrubs during the growing season is also important for understanding the water cycle in such ecosystems (Li *et al*. 2007). Transpiration is a key hydrological parameter and can also reduce run‐off. Water is first absorbed by the roots, transported through the stem and released as vapour through the leaves. Plant transpiration can thus modulate the degree to which precipitation translates into run‐off (Lesk *et al*. 2025). This effect is particularly relevant in mountain regions because of topography and often increasing precipitation with elevation.

#### Sap flow

Only a few studies have analysed patterns of transpiration in mountain shrubs by measuring sap flow rates (i.e. the upward movement of the xylem sap; Sala *et al*. 1996; Fisher *et al*. 2007; Gonzalez‐Rodriguez *et al*. 2017; Dudley *et al*. 2018; van den Bergh *et al*. 2018; Dharmadi *et al*. 2022; Bailey *et al*. 2022; Skelton *et al*. 2023). Overall, radiation, vapour pressure deficit, and leaf area index (LAI; Hu *et al*. 2008) were found to be the main determinants of sap flow.

Two studies compared the sap flow rates of shrubs and trees in the same habitat. Dharmadi *et al*. (2022) showed that the shrub *Rhododendron maximum* and the co‐occurring (diffuse‐porous) trees with comparable stem diameters had similar values of daily sap flow in the southern Appalachian Mountains. Fisher *et al*. (2007) measured nocturnal sap flow in the shrubs *Arctostaphylos manzanita* and *Ceanothus cordulatus* and in co‐occurring trees in the Sierra Nevada mountains and found that the water used by the shrubs during the night was only marginal compared to that of the trees.

A seasonal trend in sap flow was reported by Gonzalez‐Rodriguez *et al*. (2017) and Dudley *et al*. (2018), with similar results for the endemic shrub species *Spartocytisus supranubius* growing in Tenerife and *Discaria toumatou* growing in New Zealand. The highest sap flow rates were found in late spring and early summer, with a progressive decrease towards the end of summer, which followed decreasing water availability. van den Bergh *et al*. (2018) scaled the sap flow rates of individual *Alnus* shrubs in the Alps to canopy level, revealing that evapotranspiration rates of the shrub canopy were 31% higher than those of a nearby grassland. Moreover, these differences increased with higher vapour pressure deficits, with consequences for surface runoff and canopy surface temperatures, which decreased with increasing transpiration (van den Bergh *et al*. 2018).

#### Stomatal and cuticular transpiration

Sap flow in the plant is driven by stomatal and cuticular transpiration. While the first can modify the plant water relations in the short term by the stomatal response to atmospheric vapour pressure deficit and soil moisture availability, the second is influenced by cuticular properties and determines plant dehydration rates after stomatal closure (Duursma *et al*. 2019).

Stomatal conductance of two shrubs in a tropical montane forest environment (*Lantana camara* L. and *Rubus moluccanus* L.; 990 m a.s.l.) was strongly influenced by soil moisture conditions, and considerably stronger than in the co‐occurring tree species (*Psidadia altissima*; Ghimire *et al*. 2018). Accordingly, the differences in stomatal conductance between a wet and a dry period were much higher in shrubs than in trees growing at the study site (400 compared to 125 mmol m^−2^ s^−1^). Three studies (Knapp & Smith 1989 and DeLucia & Schlesinger 1991 from a Steppe climate; van den Bergh *et al*. 2018 from a Tundra climate) reported higher maximum stomatal conductance values in shrubs compared to trees. This resulted in more negative xylem water potentials in the late growing season (Knapp & Smith 1989) and lower water‐use efficiency (DeLucia & Schlesinger 1991) in shrubs. Similarly, van den Bergh *et al*. (2018) measured higher maximum conductance in *Alnus viridis* (404 ± 33 mmol m^−2^ s^−1^) than the mean value observed for tree species globally (218 ± 24 mmol m^−2^ s^−1^; Körner 1995) as well as the mean for trees retrieved from the Xylem Functional Traits Database (223 ± 0.012 mmol m‐2 s^−1^; tree species mainly from South America and China, followed by North America and Europe, and others from Oceania; Gleason *et al*. 2016).

In shrubs, decoupling from atmospheric conditions through strict stomatal regulation (i.e. isohydric behaviour) may be advantageous under short‐term drought, as seen, for example, in *Espeletia schulzii* (Sandoval *et al*. 2019). However, this behaviour may significantly reduce assimilation during prolonged drought, and strategies may depend on the shrub family (shown, e.g. by West *et al*. 2012), as well as on the microclimate conditions, such as between south‐ and north‐facing slopes (Lipscomb & Nilsen 1990). Some species can maintain a relatively high stomatal conductance during droughts, possibly because they have access to deep water through deep roots, such as *Artemisia tridentata* in the semiarid Sierra Madre Mountain (Naithani *et al*. 2012) and *Prosopis glandulosa* (de Soyza *et al*. 2004). However, most shrubs have been found to reduce transpiration during seasonal dry periods, such as desert shrubs (e.g. Smith *et al*. 1995; Kropp & Ogle 2015) and Mediterranean species during summer (Flexas *et al*. 2001; Karatassiou *et al*. 2022). Decreases in stomatal conductance of around 50% have also been documented at high elevations during cloud cover, which is very common in alpine and subalpine regions (Knapp & Smith 1987). Cui *et al*. (2016) suggested that stomatal size is important, and that smaller stomata may adjust faster than larger ones.

A few studies from Alpine regions (i.e., Erschbamer *et al*. 1983; Gerdol *et al*. 2000; Ganthaler & Mayr 2015; Anadon‐Rosell *et al*. 2017) investigated stomatal regulation of dwarf shrubs and reported large species‐specific differences. For example, stomatal regulation in *Artemisia alba* was very responsive to changes in the vapour pressure deficit, radiation, and temperature, while the two species *Helianthemum nummularium* subsp. *obscurum* and *Teucrium chamaerdrys*, growing in the same microhabitat, showed only weak stomatal control (Erschbamer *et al*. 1983). A comparison of two *Vaccinium* species, the evergreen *V. vitis‐idaea* and the deciduous *V. myrtillus*, showed no significant differences in the seasonal pattern of stomatal conductance, but lower values at the beginning of the growing season and a progressive increase towards August (Gerdol *et al*. 2000). However, *V*. *uliginosum* showed a more pronounced decrease in stomatal conductance in response to experimental drought than *V. myrtillus* (Anadon‐Rosell *et al*. 2017). In general, *Vaccinium* species seem to have risky stomatal behaviour (anisohydric strategy, i.e. they do not close stomata before the onset of xylem cavitation; Ganthaler & Mayr 2015).

Stomatal regulation can vary with elevation (e.g. Sharma *et al*. 2020) or between successional stages of the ecosystem. For example, in the shrub *Artemisia tridentata* var. *vaseyana*, stomatal limitation of photosynthesis was higher at 2135 m than at 2835 m a.s.l. (ca. 23%; Reed & Loik 2016). In contrast, shrubs from a tropical montane ecosystem showed distinct stomatal control depending on the successional stage in fallow fields, with early and intermediate species having the highest stomatal conductance and late species being more conservative (Llambí *et al*. 2003).

Unlike stomatal conductance, the cuticular conductance of mountain shrubs has been little studied. Therefore, we measured the cuticular conductance (g_min_, mmol m^−2^ s^−1^) on leaves of five selected evergreen shrub and dwarf shrub species (*Arctostaphylos uva‐ursi*, *Calluna vulgaris*, *Kalmia procumbens*, *Rhododendron ferrugineum*, *Vaccinium vitis‐idaea*; Table 3) from Mt. Patscherkofel (Tyrol, Austria) following the protocol of Sack & Scoffoni (2010). Overall, cuticular transpiration values ranged from a minimum of 0.9 (*V. vitis‐idaea*) to a maximum of 11.2 mmol m^−2^ s^−1^ (*C. vulgaris*), with an average of 4.4 ± 0.5 mmol m^−2^ s^−1^. In a meta‐analysis on leaf cuticular conductance from different species groups, Schuster *et al*. (2017) reported similar values for the two dwarf shrubs *Helianthemum appenninum* (4.07 mmol m^−2^ s^−1^) and *Teucrium chamaedrys* (1.2 mmol m^−2^ s^−1^), while other woody species had lower mean values (i.e. 1.9 mmol m^−2^ s^−1^ for the deciduous angiosperms). In addition, the mean cuticular conductance for the order Ericales, as reported by Duursma *et al*. (2019), was about 4 mmol m^−2^ s^−1^, mirroring the average found in this study, where all species belonged to Ericales.

**Table 3:** Mean leaf cuticular conductance (g_min_, mean ± SE) for five evergreen Alpine shrub species.

species	g_min_ (mmol m^−2^ s^−1^)
*Arctostaphylos uva‐ursi*	4.8 ± 0.8 b
*Calluna vulgaris*	8.97 ± 0.68 a
*Kalmia procumbens*	4.6 ± 0.44 bc
*Rhododendron ferrugineum*	2.38 ± 0.22 cd
*Vaccinium vitis‐idaea*	1.37 ± 0.09 d

Statistically significant differences between the species (*P* < 0.05) are indicated with different letters. Significant differences were tested using a One‐way ANOVA followed by a Tukey post‐hoc test in R (R Core Team 2021).

### Drought stress during the vegetation period

**Fig. 6:**
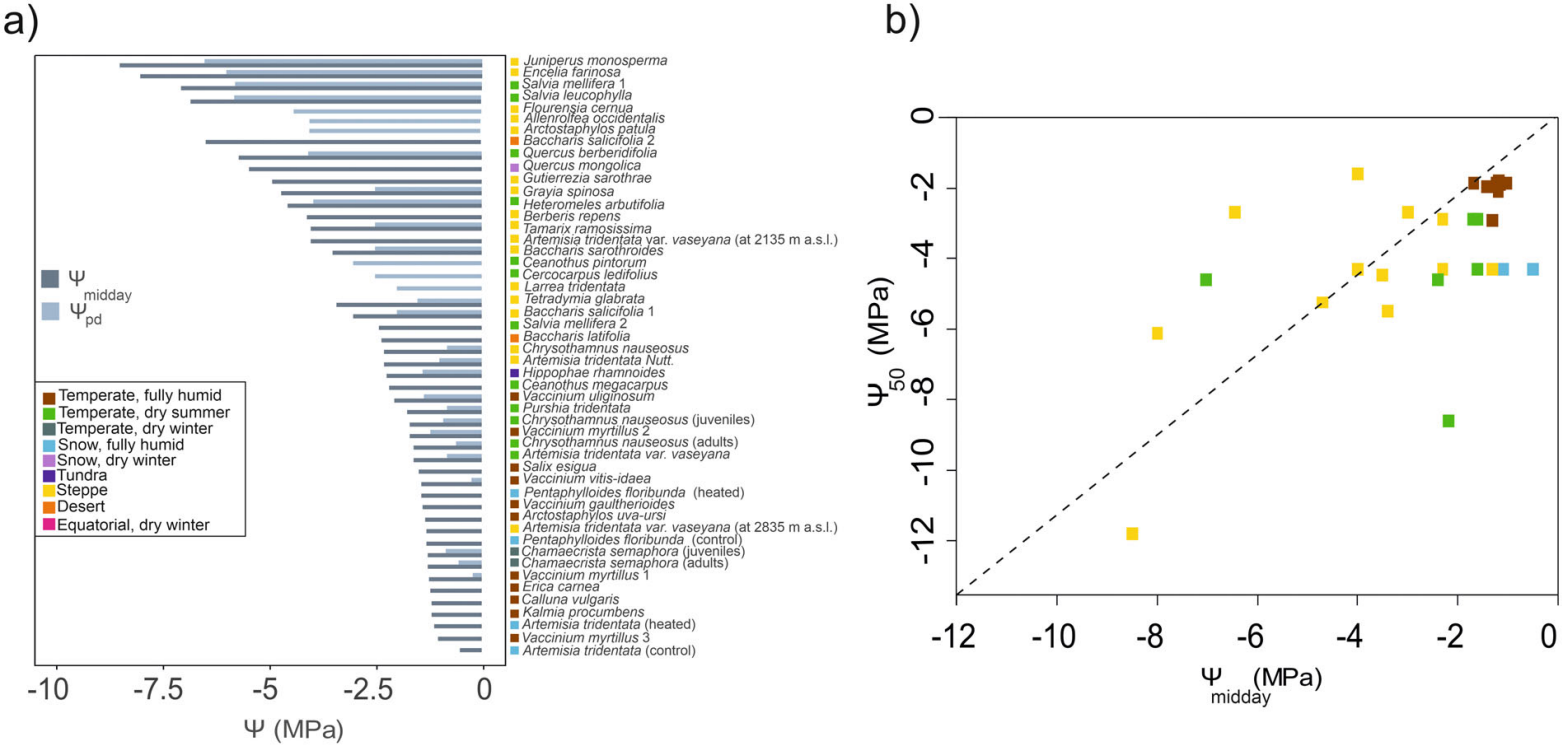
(a) Midday (Ψ_midday_) and predawn water potential (Ψ_pd_) for all mountain shrub species found in the literature search. For some shrubs only Ψ_pd_ or Ψ_midday_ were available. Shrubs found in more than one publication are indicated with numbers. Where present, different treatments, development stages, or elevations are mentioned in brackets. Coloured boxes indicate the Köppen‐Geiger climate classification (see Fig. 2; no Ψ_pd_ and Ψ_midday_ values were available for the Equatorial, dry winter climate). (b) Water potential at 50% loss of conductivity (Ψ_50_) plotted *versus* midday water potential (Ψ_midday_) for 28 shrub species. The distance from the 1:1 line indicates the safety margin, with negative values for points above it.

As there was no trend in changing mean annual precipitation with respect to elevation in the analysed dataset (Fig. 2), and to reflect the differences in drought stress experienced by the shrubs between climate zones, we summarise below the midday plant water potential (Ψ_midday_) and the predawn water potential (Ψ_pd_) of shrubs in (i) arid, (ii) warm temperate, and (iii) snow climates during and after dry periods (Fig. 6a). Interestingly, calculation of the safety margin (i.e. the difference between Ψ_midday_ and Ψ_50_, assuming Ψ_midday_ to be the minimum Ψ measured in the field; Choat *et al*. 2012) revealed that most species operate near the Ψ_50_, and five out of 28 shrub species even had a negative safety margin (Fig. 6b). This high risk of hydraulic failure might be compensated by recovery processes, for which the small size of shrubs could be an advantage. In addition, the redundancy of multiple stems is beneficial, as some of the stems may survive under drought (Ewers *et al*. 2007).

- *Arid climate*: Three of the 11 studies conducted in arid regions examined water stress along elevational gradients. Pockman & Sperry (2000) showed that most upland desert shrubs in the Sonoran Desert had lower Ψ_pd_ than riparian desert shrubs. Similar to a previous study (Halvorson & Patten 1974), Reed & Loik (2016) analysed a mountain region where precipitation increased with elevation and observed an increase in Ψ_midday_ of *Artemisia tridentata* var. *vaseyana* from −4 MPa at 2135 m to −1.3 MPa at 2835 m a.s.l. This shrub species has also been shown to exhibit a distinct isohydric behaviour, resulting in moderate differences between Ψ_pd_ and Ψ_midday_ despite the arid conditions (Prieto & Ryel 2014). Notably, several shrubs from arid mountain regions are classified as drought‐deciduous and shed their leaves during the dry season to survive periods of low water availability (de Soyza *et al*. 1996; Pockman & Small 2010; Lambers & Oliveira 2019). For example, two of these drought‐deciduous shrubs (*Grayia spinosa* and *Tetradymia glabrata*) showed a progressive decrease in Ψ_midday_ until the end of July (with minima of ca. −5 MPa and −4 MPa, respectively), while the co‐occurring evergreen *Chrysothamnus nauseosus* remained relatively constant at −2.3 ± 0.1 MPa (Branson *et al*. 1976; Hacke *et al*. 2000). The lowest Ψ_midday_ found in studies from arid mountain regions was −8.5 MPa, reported for *Juniperus monosperma* (see Fig. 6a), followed by −8 MPa for *Encelia farinosa* (Pockman & Sperry 2000), −6.45 MPa for *Baccharis salicifolia* (Rundel *et al*. 2002), −5.6 MPa for two *Quercus* spp. (Pezner *et al*. 2020; Dai *et al*. 2022), and −4.9 MPa reported for the subshrub *Gutierrezia sarothrae* (Wan *et al*. 1996). The Ψ_pd_ ranged from −6.5 (i.e. *Juniperus monosperma*; Fig. 6a) to −1.3 MPa (i.e. *Artemisia tridentata*), with intermediate values of −4.4 MPa for *Flourensia cernua* at the end of July (de Soyza *et al*. 2004), about −4.0 MPa for *Allenrolfea occidentalis* at the end of the growing season (Trent *et al*. 1997), and −2.0 MPa for *Larrea tridentata* (Castellanos‐Pérez *et al*. 2008)
- *Warm temperate climate*: Many studies from regions with warm temperate climate and dry summers have investigated plant responses to drought stress. Royce & Barbour (2001) observed a continuous decrease in Ψ_pd_ towards the end of summer in *Arctostaphylos patula* (ca. −4 MPa), *Ceanothus pintorum* (ca. −3 MPa), and *Cercocarpus ledifolius* (ca. −2.5 MPa) in the Sierra Nevada, California. Similarly, *Salix esigua* showed a decrease in midday water potential as the growing season progressed (from −0.4 ± 0.1 MPa in May to −1.48 ± 0.3 MPa in mid‐September; Bailey *et al*. 2022). In particular, Donovan & Ehleringer (1992), analysing *Chrysothamnus nauseosus*, found higher stress levels in juveniles compared to adult shrubs at the beginning of summer, with a Ψ_pd_ of −0.9 ± 0.2 and −0.6 ± 0.09 MPa and a Ψ_midday_ of −1.7 ± 0.2 and 1.6 ± 0.2 MPa in juveniles and adults, respectively. Regarding the differential effect of drought on understorey shrubs and tree survival, Lloret *et al*. (2004) documented more severe effects on trees (e.g. *Quercus ilex*) compared to shrubs (e.g. *Erica arborea*), attributing this difference to the high resprouting capacity of shrubs. Similarly, Koepke *et al*. (2010) reported that while both trees and shrubs were affected by drought, shrubs exhibited higher canopy dieback. This dieback allows shrubs to sacrifice some stems without causing whole plant mortality, enabling growth to continue after the drought subsides. The reported Ψ_pd_ ranged from −0.8 MPa (*Artemisia tridentata* and *Purshia tridentata* from the Sierra Nevada; Loik *et al*. 2015) to −5.8 MPa (*Salvia leucophylla* and *S. mellifera* from the Santa Monica Mountains of California; Pezner *et al*. 2020). The Ψ_midday_ varied from ca. −2.0 MPa (*Salvia mellifera* and *Ceanothus megacarpus* from the California Coastal Ranges; Kolb & Davis 1994) to minimum values of −4.5 MPa and −7.0 MPa in *Heteromeles arbutifolia* and *Salvia mellifera*, respectively (Pezner *et al*. 2020). No consistent differences were reported between evergreen and deciduous shrub species. In a warm temperate with dry winter climate, Ψ_pd_ was lower for young *Chamaecrista semaphora* than for adult shrubs, while Ψ_midday_ varied between −1 and −1.5 MPa for both young and adult shrubs (Castro *et al*. 2016). In a warm temperate fully humid climate, three studies (Gerdol *et al*. 2004; Anadon‐Rosell *et al*. 2017; Ganthaler & Mayr 2021) focussed on the summer response of Alpine dwarf shrubs. In mid‐July, Gerdol *et al*. (2004) measured average Ψ_pd_ values of −0.22 and −0.24 MPa and a Ψ_midday_ of −1.22 and −1.42 MPa for *Vaccinium myrtillus* and *V. vitis‐idaea*, respectively. Anadon‐Rosell *et al*. (2017) also observed only mild water stress in *Vaccinium* shrubs during a typical summer drought period. However, Ψ_pd_ and Ψ_midday_ dropped to lower values in *V. uliginosum* than in *V. myrtillus*, indicating different stress levels between species. Ganthaler & Mayr (2021) reported Ψ_midday_ for six Alpine dwarf shrub species after a typical summer drought period with values between −1.4 ± 0.05 MPa (*V*. *gaultherioides*) and −1.0 ± 0.03 MPa (*V. myrtillus*).
- *Snow climate*: Only a small number of the studies reviewed were conducted in regions with a snow climate. Dai *et al*. (2022) found a lower Ψ_midday_ for *Quercus mongolica* growing as a shrub (−5.5 MPa) than growing as a tree (−3.6 MPa) in July. Similarly, Smith (1981) reported that the dwarf shrub *Berberis repens* reached a minimum Ψ_midday_ of −4.1 MPa. An *in‐situ* climate change experiment by Shaw *et al*. (2000) reported Ψ_midday_ of −0.5 and −1.3 MPa in controls and −1.1 and −1.4 MPa in heated plants of *Artemisia tridentata* and *Pentaphylloides floribunda*, respectively.

### Winter stress

Winter conditions in temperate and tropical mountain areas can be challenging for shrubs. When snow cover is absent, low temperatures can block water uptake as the soil freezes while transpiration continues, especially on sunny days driven by large temperature gradients between the plants and atmosphere (Mayr *et al*. 2019), causing water stress. These conditions cause steep water potential gradients within the plant and can lead to frost drought (also called frost desiccation; Tranquillini 1982), and to excess xylem embolism (Mayr *et al*. 2002, 2006, 2020), threatening shrub survival. In addition to frost drought, freeze–thaw cycles, which can occur frequently at higher elevations, can also lead to embolism (Lipp & Nilsen 1997). Furthermore, low temperatures, mechanical damage (e.g. by wind, ice blast, snow loads), and the duration of the long winter period, can affect the vitality of shrubs. Snow cover, in contrast, can provide protection by buffering soil and plant temperatures and improving water status after snowmelt (Mayr & Charra‐Vaskou 2007). However, earlier snowmelts, which are becoming more likely in mountain areas worldwide, may expose shrubs to spring frosts (Lambrecht *et al*., 2007). Below, we summarise the findings on winter stress in shrubs, mainly from temperate regions, with a brief insight from tropical mountains.

Mayr *et al*. (2012) reported that the water potential of three shrub species from the Alps was significantly improved in snow‐covered sites compared to snow‐free sites. Another study (Loik *et al*. 2015) also highlighted the effects of snow depth on various physiological parameters, such as stem water potential, stomatal conductance, and CO_2_ assimilation, in a 10‐year experiment on *Artemisia tridentata* var. *vaseyana* and *Purshia tridentata*. Larcher & Siegwolf (1985) documented pronounced effects of frost drought on *Rhododendron ferrugineum* in the Alps. Reduced snow cover following an experimental snow manipulation led to a decrease in shrub water potential after only a few days, suggesting rapid desiccation in the absence of snow protection. Similarly, Mayr *et al*. (2019) found that the shrub *Pinus mugo* L. can be highly embolized in winter, especially when branches are only protected by snow for a short time and because shrubs have lower water storage capacity than trees to buffer water loss. Feild & Brodribb (2001) analysed freeze–thaw induced embolism in a treeline heath and found that gymnosperms experienced lower conductivity loss than angiosperms (which have wider conduits). Cordero & Nilsen (2002) found that even a single freeze–thaw cycle can cause a significant loss of conductivity in *Rhododendron* species. In addition to the number of freeze–thaw cycles, temperature may also influence embolism formation. This is supported by Pockman & Sperry (1996), Langan *et al*. (1997), and Martínez‐Vilalta & Pockman (2002).

Winter conditions in a tropical mountain area (Andean Cordillera of northern Chile) were reported by Rundel *et al*. (2002). In this area, winter is the dry season, and temperatures are significantly lower than in summer, which is the wet season. The Ψ_midday_ was similar between two (drought‐deciduous) subshrubs (*Baccharis salicifolia* and *Coreopsis suaveolens*) and two (evergreen) shrubs (*Chuquiraga spinosa* and *Fabiana densa* var. *ramulosa*), ranging from −6.5 to −7.3 MPa. These values of Ψ_midday_ found for mountain shrubs may reflect the arid climate of the study area, which receives an average of 160 mm of precipitation per year.

## CONCLUSIONS AND FUTURE PERSPECTIVES

The aims of this study were to analyse the hydraulic traits of mountain shrubs across various climates and regions, and to identify patterns and trade‐offs in their water relations. Based on the available literature (and additional measurements of cuticular conductance of selected shrub species), a comprehensive overview on the hydraulic strategies of mountain shrubs and their global variations is provided.

### General findings

Within the mountain shrubs dataset collected for this review, the warm temperate climate was the most represented (about 46% of publications), followed by the arid (about 28%), the snow (15%), polar (10%), and the equatorial (1%) climates. Most studies were carried out in the North American Cordillera, in the Alps, and in the Qinghai‐Tibetan Plateau. Elevation ranged from 126 m a.s.l. in the coastal region of the North American Cordillera (Kolb & Davis 1994) to 4500 m a.s.l. in the Qilian Mountains (i.e. northern part of the Qinghai‐Tibet Plateau; Liu *et al*. 2015). The diversity of mountain areas studied reflects the world's shrub‐dominated ecosystems, such as tropical alpine fields, cold deserts, Mediterranean ecosystems, and alpine tundra, although not all were equally and sufficiently represented (see below).

Overall, we found a broad range in *k*_s_ for the species studied, especially at about 400 mm mean annual precipitation (Fig. 5a), and tundra, snow with dry winter, and steppe species had distinctly higher values than species in temperate and snow fully humid climates (Table 2). The dataset thus does not correspond to the globally observed higher hydraulic efficiency in habitats with higher precipitation (and higher temperature; He *et al*. 2020). No clear trend between *k*_s_ and elevation was observed (Fig. 5c; *R* ^2^ = 0.14). Concerning hydraulic safety, we observed a wide range of Ψ_50_ at lower mean annual precipitation, possibly indicating alternative strategies to achieve safety (i.e., stomatal regulation), while higher vulnerability (less negative Ψ_50_) seems to be associated with habitats with more precipitation (Fig. 5b). Many species had comparably low or even negative safety margins, which may indicate that recovery processes play an important role (Fig. 6). Again, we found no clear trend between Ψ_50_ and elevation (Fig. 5d; *R* ^2^ = 0.22). For shrubs from temperate fully humid regions, the mean Ψ_50_ values were less negative than those for species from temperate dry summer conditions (Table 2). Compared to average values available for trees, we found that the analysed mountain shrubs had lower mean *k*_s_, with deciduous angiosperm shrubs having the highest values. In terms of xylem hydraulic safety, shrubs appear to be more resistant than trees, with deciduous angiosperm shrubs having lower resistance than evergreen angiosperms and conifers. However, as the number of studies was limited, general conclusions on differences between shrubs and trees are difficult. For dwarf shrubs, studies were even limited to the European Alps, but they found both low hydraulic efficiency and safety for this growth form.

### Limitations

This review of the currently available literature revealed that several aspects of water relations in mountain shrubs are still poorly understood. The number of studies per trait was limited, and the studies were not evenly distributed between climate zones and altitudinal ranges, making comparisons between climate zones and with trees difficult. There is an imbalance in the data and a need for more studies, particularly in Asian and African mountain regions. For dwarf shrubs, studies were limited to the European Alps, which limits the generalisability of the results. In addition, there is very little information on water storage capacity (only one publication, Liu *et al*. 2015), the partitioning of water between storage and transpiration, or the role of water storage under drought. Similarly, cuticular conductance has received less attention than stomatal conductance and stomatal regulation.

### Future perspectives

Considering water uptake, further analysis of plant morphology (e.g. root‐to‐shoot ratio, maximum rooting depth) and root function is needed. Regarding water transport, we still lack a comprehensive picture of xylem anatomical characteristics in mountain shrubs, their impact on transport capacity, and trade‐offs with additional xylem functions, such as mechanical support and storage capacity. Recovery from drought‐induced embolism may also play a central role in these low‐stature plants but was rarely studied. Further analysis of shrub hydraulics could provide important new insights into plant structure–function relationships and improve our understanding of the adaptations of this widespread growth form to the environmental stresses present in mountain regions.

## AUTHOR CONTRIBUTIONS

CM, AG and SM conceived the study; CM and SM performed the literature search; CM analysed the data and wrote the manuscript with contributions from all authors. All authors agreed on the final version of the manuscript.

## Supporting information


**Table S1.** Full list of publications included in this review. For each publication hydraulic aspects considered and study species are given.
