# Supplementary material for: Global variability in hydraulic traits and water use strategies of mountain shrubs and dwarf shrubs
Source: Plant Biol (Stuttg). 2025 Sep 3;27(7):1253–70. doi: 10.1111/plb.70088 (PMC12631516; doi:10.1111/plb.70088)
Supplement: Supplementary file 1 — Table S1. Full list of publications included in this review. For each publication hydraulic aspects considered and study species are given. [file PLB-27-1253-s001.docx]

**Table S1.** Full list of publications included in this review. For each publication hydraulic aspect dealt with and the study species are given.

| **Publication** | **Water uptake** | **Water transport** | **Transpiration** | **Summer** | **Winter stress** | **Species** |
| --- | --- | --- | --- | --- | --- | --- |
| Anadon-Rosell et al., 2017 |  |  | x | x |  | *Vaccinium myrtillus;*  *Vaccinium uliginosum* |
| Bailey et al., 2022 | x |  | x | x |  | *Salix esigua* |
| Beikircher & Mayr, 2008 |  | x |  |  |  | *Juniperus communis* |
| Bodé et al., 2020 | x |  |  |  |  | *Rhus natalensis;*  *Protea caffra var. kilimanjarica;*  *Erica trimera;*  *Euryops dacrydioides* |
| Branson et al., 1976 |  |  |  | x |  | *Grayia spinosa;*  *Tetradimia spinosa;*  *Chrysothamnus nauseosus* |
| Castellanos-Pérez et al., 2008 |  |  |  | x |  | *Larrea tridentata* |
| Castro et al., 2016 |  |  | x | x |  | *Chamaecrista semaphora* |
| Chimner & Cooper, 2004 | x |  |  |  |  | *Sarcobatus vermicultaus;*  *Chrysothamnus nauseosus* subsp. *consimilis;*  *Chrysothamnus greenei* |
| Cordero & Nilsen, 2002 |  | x |  |  | x | *Rhododendron maximum;*  *Rhododendron catawbiense;*  *Rhododendron macrophyllum* |
| Cui et al., 2016 |  |  | x |  |  | *Syringa oblata*;  *Syringa pinnatifolia* |
| Dai et al., 2022 |  |  |  | x |  | *Quercus mongolica* |
| Darrouzet-Nardi et al., 2006 | x |  |  |  |  | *Artemisia rothrockii* |
| Davis et al., 1999 |  | x |  |  |  | *Ceanothus megacarpus*;  *Ceanothus spinosus*;  *Ceanothus cuneatus*;  *Ceanothus oliganthus*;  *Ceanothus crassofolius*;  *Ceanothus leucodermis* |
| de Soyza et al., 1996 |  |  |  | x |  | *Prosopis glandulosa* |
| de Soyza et al., 2004 |  |  |  | x |  | *Brickellia laciniata*  *Chilopsis linearis;*  *Fallugia paradoxa;*  *Prosopis glandulosa;*  *Rhus microphylla;*  *Flourensia cernua* |
| Delucia & Schlesinger, 1991 |  |  | x |  |  | *Artemisia tridentata*;  *Amelanchier alnifolia*;  *Purshia tridentata*;  *Arctostaphylos patula* |
| Dharmadi et al., 2022 |  |  | x |  |  | *Rhododendron maximum* |
| Dodd et al., 1998 | x |  |  |  |  | *Atriplex canescens* |
| Donovan & Ehleringer, 1992 | x |  |  |  |  | *Chrysothamnus nauseosus* |
| Donovan & Ehleringer, 1994 | x |  |  |  |  | *Artemisia tridentata*;  *Chrysothamnus nauseosus*;  *Chrysothamnus viscidiflorus*;  *Gutierrezia sarothrae*;  *Juniperus osteosperma*;  *Tetradymia canescens* |
| Duan et al., 2008 | x |  |  |  |  | *Nitraria tangutorum* |
| Dudley et al., 2018 | x |  | x |  |  | *Discaria toumatou* |
| Erschbamer, Grabherr & Reisgl, 1983 |  | x |  |  |  | *Artemisia alba;*  *Helianthemum nummularium spp. obscurum;*  *Teucrium chamaedrys* |
| Feild & Brodribb, 2001 |  |  |  |  | x | *Diselma archeri;*  *Podocarpus lawrencei; Leptospermum rupestre*;  *Orites revoluta*;  *Ozothamnus rodwayi*;  *Richea scoparia*;  *Tasmannia lanceolata* |
| Feng et al., 2022 |  | x |  |  |  | *Potentilla glabra;*  *Potentilla fruticosa;*  *Ilex pernyi;*  *Cotoneaster multiflorus;*  *Salix gilashanica;*  *Caragana jubata;*  *Lonicera hispida;*  *Spiraea alpina* |
| Fernandez & Cladwell, 1975 | x |  |  |  |  | *Atriplex confertifolia;*  *Ceratoides lanata;*  *Artemisia tridentata;* |
| Fickle et al., 2023 |  | x |  |  |  | *Arctostaphylos viscida Cercocarpus betuloides;*  *Fremontodendron californicum;*  *Garrya flavescens; Quercus garryana* |
| Fisher et al., 2007 |  |  | x |  |  | *Arctostaphylos manzanita*;  *Ceanothus cordulatus;* |
| Flexas et al., 2001 |  |  | x |  |  | *Pistacia lentiscus* |
| Franco et al., 1994 | x |  |  |  |  | *Larrea tridentata* |
| Ganhaler et al., 2022 |  | x |  |  |  | *Vaccinium myrtillus*;  *Vaccinium gaultheroides*; *Calluna vulgaris*;  *Arctostaphylos uva-ursi*;  *Kalmia procumbens*;  *Erica carnea* |
| Ganthaler & Mayr, 2015 |  | x |  |  |  | *Vaccinium myrtillus*;  *Vaccinium vitis-idaea* |
| Ganthaler & Mayr, 2021 |  | x |  | x |  | *Arctostaphylos uva-ursi.*;  *Kalmia procumbens;*  *Calluna vulgaris;*  *Erica carnea;*  *Vaccinum myrtillus;*  *Vaccinium gaultherioides* |
| Gerdol et al., 2000 |  |  | x |  |  | *Vaccinium myrtillus*;  *Vaccinium vitis-idaea* |
| Gerdol et al., 2004 |  |  |  | x |  | *Vaccinium myrtillus*;  *Vaccinium vitis-idaea* |
| Ghimire et al., 2017 |  |  | x |  |  | *Lantana camara*;  *Rubus moluccanus;* |
| Gibbens & Lenz, 2001 | x |  |  |  |  | *Flourensa cernua;*  *Larrea tridentata* |
| Gonzales-Rodriguez et al., 2017 |  |  | x |  |  | *Spartocytisus supranubius* |
| Goodwin & Hurteau, 2024 | x |  |  |  |  | *Ceanothus cordulatus;*  *Arctostaphylos patula* |
| Hacke, Sperry & Pittermann, 2000 |  | x |  |  |  | *Chrysothamnus nauseosus;*  *Chrysothamnus viscidiflorus;*  *Atriplex confertifolia;*  *Grayia spinosa;*  *Tetradymia glabrata;*  *Atriplex canescens* |
| Halvorson & Patten, 1974 |  |  |  | x |  | *Larrea tridentata;*  *Franseria deltoidea;*  *Krameria grayi;*  *Simmondsia chinensis; Eriogonum fasciculatum;*  *Haplopappus spinulosus* |
| Hu et al., 2008 |  |  | x |  |  | *Potentilla fruticosa* |
| Jacobsen & Pratt, 2014 |  | x |  |  |  | (review paper) |
| Karatassiou et al., 2022 |  |  | x | x |  | *Arbutus andrachne*; *Arbutus unedo*; *Quercus ilex*; *Quercus coccifera*; *Carpinus betulus*; *Cercis siliquastrum*; *Coronilla emeroides*; *Pistacia terebinthus* |
| Kitajima et al., 2013 | x |  |  |  |  | *Arctostaphylos glandulosa*;  *Eriogonum wrightii* |
| Knapp & Smith, 1987 |  |  | x |  |  | *Artemisia tridentata* |
| Knapp & Smith, 1989 |  |  | x |  |  | *Juniperus communis*;  *Arctostaphylos uva-ursi*;  *Berberis repens* |
| Koepke et al., 2010 |  |  |  | x |  | *Brickellia grandiflora;*  *Ceratoides lanata; Chrysothamnus nauseosus;*  *Ephedra viridis;*  *Fallugia paradoxa;*  *Forestiera neomexicana;*  *Rhus trilobata;*  *Ribes cereum;*  *Arctostaphylos pungens; Berberis fremontii;*  *Cercocarpus montanus;*  *Purshia stansburiana*  *Quercus turbinella;*  *Rhus trilobata;*  *Tetradymia canescens; Amelanchier utahensis;*  *Chamaebatiaria millefolium* |
| Kolb & Davis, 1994 |  | x |  | x |  | *Ceanothus megacarpus;*  *Salvia mellifera* |
| Kolb & Sperry, 1999 |  | x |  |  |  | *Artemisia tridentata* |
| Kropp & Ogle, 2015 |  |  | x |  |  | *Larrea tridentata* |
| Kropp et al., 2016 | x |  |  |  |  | *Larrea tridentata* |
| Langan, Ewers & Davis, 1997 |  |  |  |  | x | *Rhus laurina;*  *Ceanothus megacarpus* |
| Larcher & Siegwolf, 1985 |  |  |  |  | x | *Rhododendron ferrugineum* |
| Li et al., 2007 | x |  |  |  |  | *Potentilla fruticosa* |
| Li et al., 2019 |  |  | x |  |  | *Potentilla fruticosa* |
| Lipp & Nilsen, 1997 |  | x |  |  | x | *Rhododendron maximum* |
| Lipscomb & Nilsen, 1990 |  |  | x |  |  | *Rhododendron maximum;*  *Kalmia latifolia*;  *Rhododendron periclymenoides* |
| Liu et al., 2015 |  |  |  |  |  | *Salix cupularis;*  *Salix oritrepha;* |
| Llambì et al., 2003 |  |  | x |  |  | *Acaena elongata*;  *Baccharis prunifolia* |
| Lloret et al., 2004 |  |  |  | x |  | *Erica arborea* |
| Loik et al., 2015 |  |  |  | x | x | *Artemisia tridentata* var. *vaseyana;*  *Purshia tridentata* |
| Martinez-Vilalta & Pockman, 2002 |  | x |  |  | x | *Larrea tridentata* |
| Mayr et al., 2010 |  | x |  |  |  | *Rhododendron ferrugineum;*  *Rhododendron hirsutum;*  *Rhododendron x intermedium* |
| Mayr et al., 2019 |  |  |  |  | x | *Pinus mugo* |
| Naithani et al., 2012 |  |  | x |  |  | *Artemisia tridentata* var. *vaseyana* |
| Pezner et al., 2020 |  |  |  | x |  | *Heteromeles arbutifolia*; *Quercus berberidifolia*; *Salvia leucophylla*; *Salvia mellifera* |
| Pockman & Small, 2010 |  |  |  | x |  | *Larrea tridentata* |
| Pockman & Sperry, 1996 |  |  |  |  | x | *Larrea tridentata* |
| Pockman & Sperry, 2000 |  | x |  | x |  | *Baccharis sarothroides;*  *Baccharis salicifolia;*  *Tamarix ramosissima;*  *Encelia farinosa;*  *Juniperus monosperma* |
| Prieto & Ryel, 2014 | x |  |  | x |  | *Artemisia tridentata* |
| Redtfeldt & Davis, 1996 |  | x |  |  |  | *Adenostoma fasciculatum;*  *Adenostoma sparsifolium* |
| Reed & Loik, 2016 |  |  | x | x |  | *Artemisia tridentata* ssp. *vaseyana* |
| Rong et al., 2011 | x |  |  |  |  | *Viburnum utile;*  *Pyracantha fortuneana;*  *Rhamnus davurica* |
| Royce and Barbour, 2001 |  |  |  | x |  | *Arctostaphylos patula*;  *Cercocarpus ledifolius*;  *Ceanothus pintorum* |
| Rundel et al., 2002 |  |  |  | x |  | *Baccharis latifolia*; *Baccharis santelecis*; *Chuqiraga spinosa*; *Fabiana densa* var. *ramulosa*; *Lophopappus cuneatus*; *Lupinus oreophilus*; *Senna cumungii* var. *eremobia*; *Ademisia spinosissima*; *Baccharis salicifolia*; *Balbisia stitchkinii*; *Coreopsis suaveolens* |
| Rundel et al., 2005 |  |  |  | x |  | *Chrysothamnus viscidiflorus* subsp. *viscidiflorus;*  *Linanthus nuttallii* subsp*. pubescens* |
| Sala et al., 2006 |  |  | x |  |  | *Tamarix ramosissima* |
| Sandoval et al., 2019 |  |  | x |  |  | *Hypericum laricifolium*;  *Espeletia schultzii* |
| Schwinning et al., 2005 | x |  |  |  |  | *Gutierrezia sarothrae;*  *Ceratoides lanata;* |
| Sharma et al., 2020 |  |  | x |  |  | *Artemisia tridentata* ssp. *wyomingensis;*  *Artemisia tridentata* ssp. *vaseyana* |
| Shaw, Loik & Harte, 2000 |  |  |  | x |  | *Artemisia tridentata*;  *Pentaphylloides floribunda* |
| Shi et al., 2021 | x |  |  |  |  | *Salix oritrepha;*  *Salix cupularis;*  *Rhododendron capitatum; Rhododendron thymifolium; Spiraea salicifolia;*  *Potentilla fruticosa* |
| Skelton et al., 2023 |  | x | x |  |  | *Erica monsoniana;*  *Protea repens* |
| Smith et al., 1995 |  |  | x |  |  | *Grayia spinosa*;  *Haplopappus cooperi*;  *Hymenoclea salsola*;  *Salazaria mexicana* |
| Smith, 1981 |  |  |  | x |  | *Rosa acicularis;*  *Berberis repens* |
| Sternberg et al., 1996 | x |  |  |  |  | *Adenostoma fasciculatum;*  *Arctostaphylos glandulosa;*  *Ceanothus greggii* |
| Szutu & Papuga, 2019 | x |  | x |  |  | *Larrea tridentata* |
| Thorburn & Ehleringer, 1995 | x |  |  |  |  | *Artiplex canescens;*  *Chrysothamnus nauseosus;*  *Vanclevea stylosa* |
| Tian et al., 2023 | x |  |  |  |  | *Hippophae rhamnoides;*  *Artemisia ordosica;* |
| Trent et al., 1997 |  |  |  | x |  | *Allenrolfea occidentalis;*  *Sarcobatus vermiculatus* |
| van den Bergh et al., 2018 |  |  | x |  |  | *Alnus viridis* |
| Wan, Sosebee & McMichael, 1996 |  |  |  | x |  | *Gutierrezia sarothrae* |
| Wen et al., 2022 | x |  |  |  |  | *Eurya muricata*;  *Rhododendron dilatatum* |
| West et al., 2012 | x |  |  |  |  | *Leucadendron laureolum;*  *Erica pyxidiflora;*  *Erica ericoides;*  *Erica subcapitata* |
| Wu et al., 2016 | x |  |  |  |  | *Hippophae rhamnoides*;  *Artemisia oxycephala* |
| Wu et al., 2019a | x |  |  |  |  | *Hippophae rhamnoides* |
| Wu et al., 2019b | x |  |  |  |  | *Myricaria squamosa* |
| Yao et al., 2024 |  | x |  |  |  | *Caragana jubata;*  *Salix gilashanica* |
| Zhang et al., 2020 | x |  |  |  |  | *Caragana korshinskii*;  *Reaumuria soongorica* |
| Zhang et al., 2022 | x |  |  |  |  | *Salix cupularis*;  *Potentilla fruticosa;*  *Rhododendron thymifolium*;  *Salix oritrepha*;  *Salix sclerophylla*;  *Rhododendron anthopogonoides;*  *Rhododendron przewalskii; Caragana jubata* |
| Zhu et al., 2016 | x |  |  |  |  | *Salix psammophila*;  *Salix cheilophila* |
| Zhu et al., 2021 | x |  |  |  |  | *Ziziphus jujuba* var. *spinosa*;  *Vitex negundo* var. *heterophylla* |
|  |  |  |  |  |  |  |
|  |  |  |  |  |  |  |
|  |  |  |  |  |  |  |
|  |  |  |  |  |  |  |
|  |  |  |  |  |  |  |
|  |  |  |  |  |  |  |
|  |  |  |  |  |  |  |
|  |  |  |  |  |  |  |
|  |  |  |  |  |  |  |
|  |  |  |  |  |  |  |
|  |  |  |  |  |  |  |
|  |  |  |  |  |  |  |
|  |  |  |  |  |  |  |
|  |  |  |  |  |  |  |
|  |  |  |  |  |  |  |
|  |  |  |  |  |  |  |
|  |  |  |  |  |  |  |
|  |  |  |  |  |  |  |
|  |  |  |  |  |  |  |
|  |  |  |  |  |  |  |
|  |  |  |  |  |  |  |
|  |  |  |  |  |  |  |
|  |  |  |  |  |  |  |
|  |  |  |  |  |  |  |
|  |  |  |  |  |  |  |
